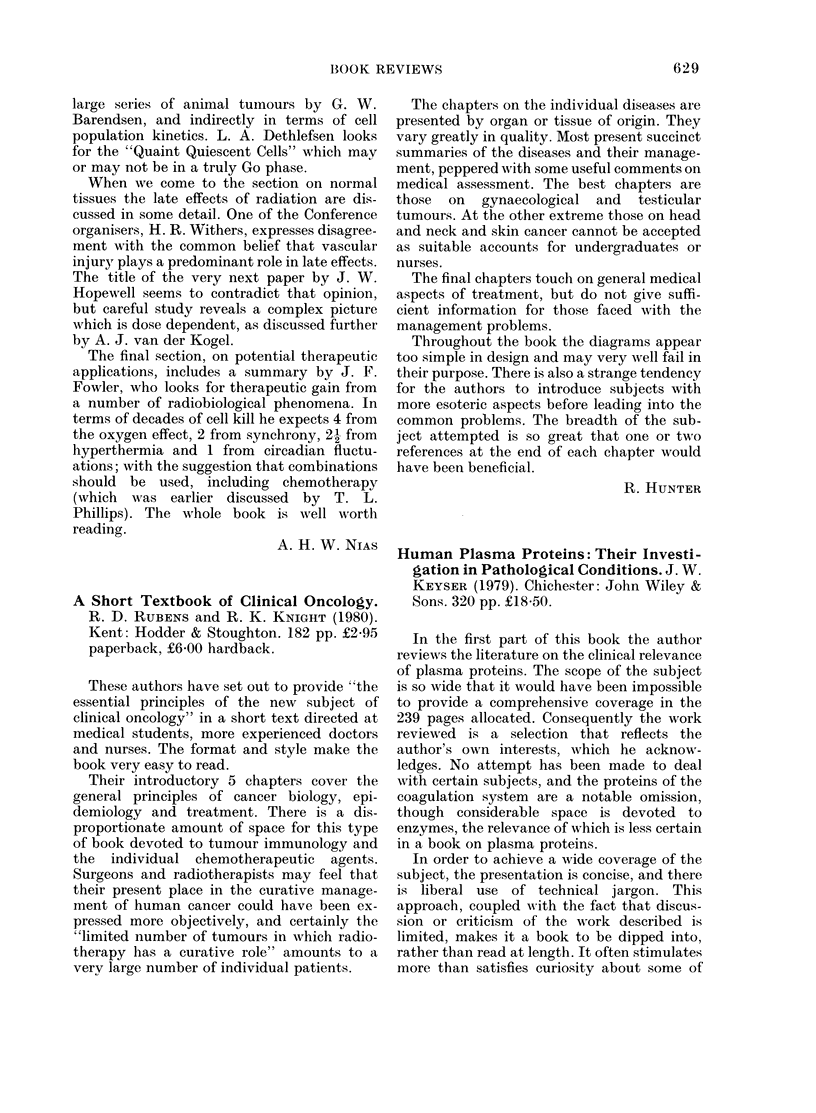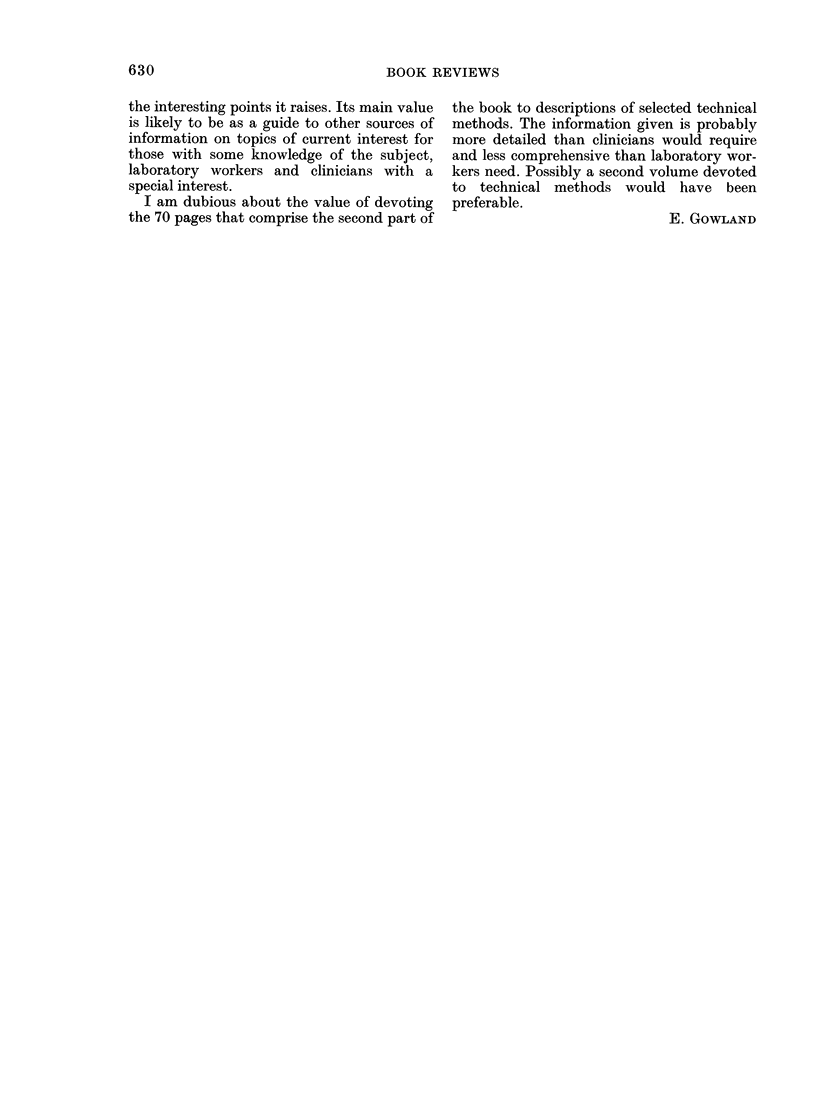# Human Plasma Proteins: Their Investigation in Pathological Conditions

**Published:** 1980-10

**Authors:** E. Gowland


**Human Plasma Proteins: Their Investigation in Pathological Conditions.** J. W. KEYSER (1979). Chichester: John Wiley & Sons. 320 pp. £18·50.

In the first part of this book the author reviews the literature on the clinical relevance of plasma proteins. The scope of the subject is so wide that it would have been impossible to provide a comprehensive coverage in the 239 pages allocated. Consequently the work reviewed is a selection that reflects the author's own interests, which he acknowledges. No attempt has been made to deal with certain subjects, and the proteins of the coagulation system are a notable omission, though considerable space is devoted to enzymes, the relevance of which is less certain in a book on plasma proteins.

In order to achieve a wide coverage of the subject, the presentation is concise, and there is liberal use of technical jargon. This approach, coupled with the fact that discussion or criticism of the work described is limited, makes it a book to be dipped into, rather than read at length. It often stimulates more than satisfies curiosity about some of the interesting points it raises. Its main value is likely to be as a guide to other sources of information on topics of current interest for those with some knowledge of the subject, laboratory workers and clinicians with a special interest.

I am dubious about the value of devoting the 70 pages that comprise the second part of the book to descriptions of selected technical methods. The information given is probably more detailed than clinicians would require and less comprehensive than laboratory workers need. Possibly a second volume devoted to technical methods would have been preferable.

E. Gowland